# Structural activity analysis, spectroscopic investigation, biological and chemical properties interpretation on Beta Carboline using quantum computational methods

**DOI:** 10.1016/j.heliyon.2019.e02788

**Published:** 2019-11-20

**Authors:** K. Hemachandran, P. Anbusrinivasan, S. Ramalingam, R. Aarthi, C.K. Nithya

**Affiliations:** a Department of Chemistry, A.V.C. College, Mayiladuthurai, Tamilnadu, India; b Department of Physics, A.V.C. College, Mayiladuthurai, Tamilnadu, India; c Department of Physics, ST. Theresas College of Arts and Science, Tharangambadi, Tamilnadu, India

**Keywords:** Organic chemistry, Theoretical chemistry, Beta carboline, QSAR, Biological activity, Chemical reaction path, Bathochromic, Electronic shift, VCD

## Abstract

In this methodological work, the structural activity analysis have been carried out on β-Carboline to study the anti cancer activity and the way of improving the biological activity. The molecular spectroscopic tools were used to evaluate all the experimental data like spectral results and data were validated by the computational, HyperChem and Osiris tools. The structural, biological and physico-chemical related analyses have been performed to interpret the properties. The GPCR ligand calculated to be 0.11 for generating pharmacokinetic process, Specified drug information for the compound, was congregated from all types of structural activity which was drawn by spectral and HyperChem data. The σ and π interaction band gap (6.18 eV) ensured the drug consistency. The Mulliken charge process distribution was mapped, the charge orientation assignment was checked; the acquired negative charge potential consignment for the cause of antibiotic impact was verified. The molecular orbital interaction study was carried out to identify the origination of degeneracy of interaction causing drug mechanism. Using NMR spectral pattern, the chemical reaction path was recognized and the nodal region dislocation was distinguished on chemical shift. The Electronegativity (χ) and Electrophilicity charge transfer found to be 3.83 and 0.215, confirmed charge complex transfer for activating drug process in the compound. The molecular nonbonding section was thoroughly observed in order to find the occupancy energy, was the key process to initiate drug activity. The bathochromic electronic shift was observed and the existence of CT complex was discussed. The hindering of toxicity was inspected on inevitable chirality of the compound by specifying VCD spectrum.

## 1 Introduction

The B-Carboline also called as β -Carboline is the combined form of pyridine and indole which belongs to the tryptamine family [1]. The compound has been isolated primarily from plants and marine creatures and it was identified due to its biological activities such as antitumor, antiviral, antiparasitic and antimicrobial activities [2, 3, 4]. β- Carboline is heterocyclic chemical and belongs to alkaloids family, are extensively found in plants and animals, and invariably acted as GABA_A_ inverse agonists [5]. Usually, the Indole derivatives are very important heterocyclic compounds which are basically having successful biological potential causing drug activity such as antibiotic [6].

The chemical species; indole derivatives have great consideration in the pharmaceutical industry as bioactive molecules against microorganisms, cancer cells and different class of disorder in the human body [7]. Since the indole is substituted with pyridine ring, the chemical reactivity of indole is altered with respect to the heterocyclic structure; pyridine. Simultaneously, the electrophilic aromatic substitution effect is taking place and thereby the resultant compound is more chemically reactive with many folds [8]. Normally, the indole is used as antimicrobial, antibacterial and antifungal agents. By the addition of pyridine ring, the fundamental drug activity is enhanced and also, some of the additional characteristics are added with product molecule, thus the present compound become biologically more reactive and the supplementary drug properties such as antitumor, antiviral and antiparasitic activity is newly produced in the compound [9, 10].

The introduction of heterocyclic groups into indole derivative drug compound may affect their physico-chemical properties, such as alter their prototypes of assimilation and toxicity. Especially, the addition of pyridine ring with indole making complex compound; Beta Carboline become effective antibiotic drug and used for the preclusion and treatment of several cancers and malaria [11, 12]. Though, the present compound has moderate anti-cancer effects, the anticancer potential can be structurally improved by addition of active substitutional groups. Hence, due to the symmetrical placement of pyridine and benzene ring around the pyrrole ring the enantiomer characteristics are supported with the molecule and compound become less toxic nature [13]. After making thorough studies on literatures and available sources, there was no work determined to interpret and explore the biological as well as structural activity regarding drug formulating properties. In this attempt, the β-Carboline substance was evaluated against biological property, physico-chemical potential for dug action and drug activity related to structural activity.

## 2 Experimental profile

### 2.1 Physical state

• The chemical compound; β-Carboline was purchased and it was checked further that, it was chemically pure and spectroscopic grade.
• The most accurate analytical chemical analysis methods were used for determining the purity of substance in terms of melting point which was found to be 199 °C and validated by NIST standards.

### 2.2 Recording details

• The FT-IR and FT-Raman spectra of the compound were recorded by FT/IR-4000 Series with Standard Wavenumber Measurement Range of 10 cm^−1^ to 6500 cm^−1^ and Optional Extended Wavenumber Range specified as 150 cm^−1^ to 6500 cm^−1^. The wavenumber accuracy was to be ±0.01 cm^−1^ as per the Literature [14]. The High-intensity ceramic source of Halogen lamp was used with DLa TGS detector.
• The ^1^H NMR and ^13^C NMR spectra were obtained Bruker 700 MHz spectrometer with magic-angle-spinning (MAS) solid state NMR probes. The signal to noise for proton on standard ethyl benzene (0.1%) sample is equivalent to >30000:1 by mass sensitivity compared to 6000:1 along with specified parametric protocol [15].
• The experimental UV-Vis spectrum was recorded from V-700 Series of UV-visible spectrophotometers (300 MHz) in the necessary range of 50 nm–800 nm, with the scanning time of 0.20 nm, using the UV-1700 series instrument [16].

## 3 Computational details

In order to get all information regarding structural activity, the structural parameters were computed by performing the computational calculations by Gaussian 16 A. 01.version software program using iMac computer [17]. The molecular structural activity and QSAR results were accomplished by the computations in HyperChem version 8.0.10 software and Molinspiration worktable for studying biological properties. The biological parametric diagram was sketched by drawing tool to completed drug assessment parameters. The Molecular electrostatic potential (MEP) and Frontier molecular orbital profile was studied by carry out the calculations using B3LYP/6-311++G(d,p) level of theory. The ^13^C and ^1^H NMR spectra were simulated and chemical shift was observed with respect to TMS (Tetra-methyl-silane) by Gauge independent atomic orbital (GIAO) using Polarizable continuum module (I-PCM). The electronic excited orbitals have been calculated and UV-Visible absorption peak was traced from assigned by selection rule.

## 4 Results and discussion

### 4.1 Structural deformation analysis

**Fig. 1:**
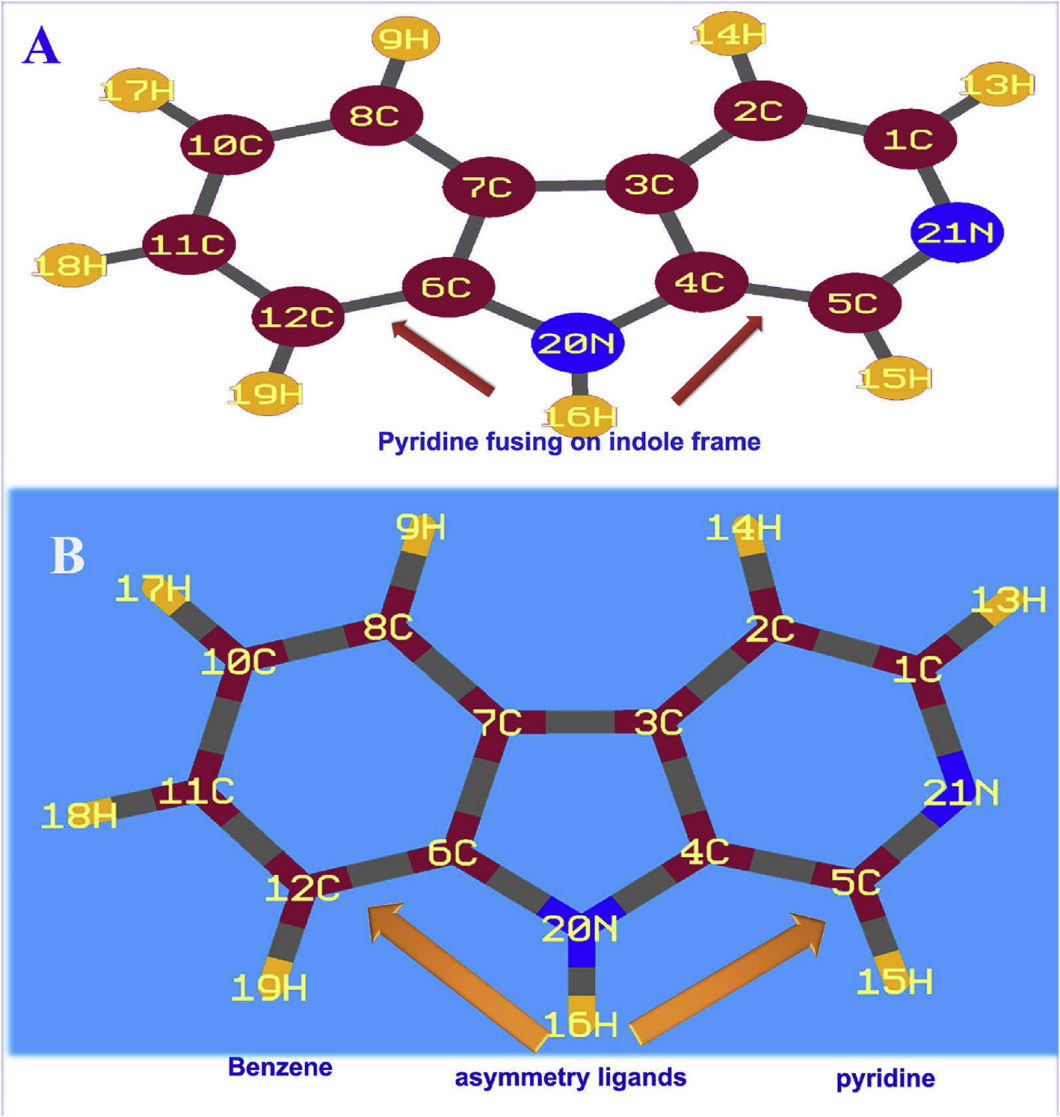
(A) Bond type (B) tube type of Molecular Structure of Beta Carboline.

The stabilized structure is portrayed in Fig. 1 and its allied structural parameters are given in Table 1. The present case was constructed by the addition of pyridine ring in indole frame and there was no further substitutions found. By adding the pyridine with indole, the asymmetricity gradient was created and it was rated within 50% in the molecule. In the core frame, since the imine group was located on C5–N21, the bond length C1–N21 was 0.015Å greater than C5–N21. Due to N atom on ring, the internuclear distance of core atoms was disturbed in the range of 0.005–0.007Å. According to the standard value [18], the bond length of C–N in pyrrole was 1.372Å. Whereas the bond length of C4–N20 and C6–N20 was same, that was found to be 1.386 Å which was very high and due to symmetrical placement of benzene and pyridine ring. The elemental bond length distance of C6–C7 and C10–C11 was calculated to be 1.419Å and 1.406Å respectively. The difference of bond length was due to the pyrrole content. From this bond length variation, it was found that, the pyridine ring substitution in the indole ring produces huge impact on the indole ring. The pyridine was a part of the main frame and due to this, ring parameters were altered with respect to the pyridine frame. The injection over the indole, the imine group was stabilized and the electronegativity was considerably increased. This view was making polarization gradient and thus negative electrochemical generated on the core frame that induced anticancer activity from antibiotic character. Similarly, the bond angle with the center of N was disturbed by 4° and the bond angle with the center of C was distorted by 2°. This was due to the ring of N was stabilized more than ring of C. The hexagonal frame of pyridine was rather disturbed than benzene which was due to the electrochemical was resided over pyridine instead of benzene ring.

**Table 1:** Optimized geometrical parameters for β-Carboline.

Geometrical Parameters	Methods				
	HF	B3LYP		B3PW91	
	6-311++G (d, p)	6-31++G (d, p)	6-311++G (d, p)	6-31++G (d, p)	6-311++G (d, p)
**Bond length(Å)**					
C1–C2	1.38	1.39	1.39	1.39	1.39
C1–H13	1.08	1.09	1.09	1.09	1.09
C1–N21	1.33	1.35	1.35	1.35	1.34
C2–C3	1.39	1.40	1.40	1.40	1.40
C2–H14	1.08	1.09	1.08	1.09	1.09
C3–C4	1.39	1.42	1.42	1.42	1.41
C3–C7	1.45	1.45	1.45	1.45	1.44
C4–C5	1.39	1.40	1.40	1.40	1.39
C4–N20	1.38	1.39	1.39	1.38	1.38
C5–H15	1.08	1.09	1.09	1.09	1.09
C5–N21	1.31	1.34	1.33	1.33	1.33
C6–C7	1.40	1.42	1.42	1.42	1.42
C6–C12	1.39	1.40	1.40	1.40	1.39
C6–N20	1.38	1.39	1.39	1.38	1.38
C7–C8	1.39	1.40	1.40	1.40	1.40
C8–H9	1.08	1.09	1.08	1.09	1.09
C8–C10	1.38	1.39	1.39	1.39	1.39
C10–C11	1.40	1.41	1.41	1.41	1.40
C10–H17	1.08	1.09	1.08	1.09	1.09
C11–C12	1.38	1.39	1.39	1.39	1.39
C11–H18	1.08	1.09	1.08	1.09	1.09
C12–H19	1.08	1.09	1.08	1.09	1.09
H16–N20	0.99	1.01	1.01	1.01	1.01
**Bond angle(º)**					
C2–C1–N21	122.1	121.9	121.9	121.9	121.9
H13–C1–N21	118.0	117.7	117.7	117.7	117.7
C1–C2–C3	135.5	135.6	135.6	135.6	135.6
C1–C2–H14	106.5	106.7	106.7	106.7	106.7
C3–C2–H14	120.1	120.4	120.3	120.3	120.3
C2–C3–C4	109.2	108.8	108.8	108.8	108.8
C2–C3–C7	130.7	130.8	130.9	130.9	130.9
C4–C3–C7	121.6	121.7	121.7	121.7	121.7
C3–C4–C5	121.2	121.2	121.2	121.3	121.3
C3–C4–N20	117.3	117.1	117.1	117.0	117.1
C5–C4–N20	121.5	121.7	121.7	121.7	121.7
C4–C5–H15	109.2	108.7	108.7	108.8	108.7
C4–C5–N21	129.3	129.5	129.6	129.5	129.6
H15–C5–N21	106.3	106.5	106.6	106.5	106.5
C7–C6–C12	133.9	134.0	134.0	134.0	134.0
C7–C6–N20	119.8	119.5	119.5	119.5	119.5
C12–C6–N20	120.6	120.5	120.5	120.5	120.5
C3–C7–C6	119.1	119.1	119.1	119.0	119.0
C3–C7–C8	120.4	120.5	120.4	120.5	120.5
C6–C7–C8	120.4	120.7	120.7	120.7	120.7
C7–C8–H9	120.1	119.9	119.9	119.9	119.9
C7–C8–C10	119.5	119.5	119.4	119.5	119.4
H9–C8–C10	121.7	121.5	121.5	121.5	121.5
C8–C10–C11	122.1	121.9	121.9	121.9	121.9
C8–C10–H17	118.0	117.7	117.7	117.7	117.7
C11–C10–H17	135.5	135.6	135.6	135.6	135.6
C10–C11–C12	106.5	106.7	106.7	106.7	106.7
C10–C11–H18	120.1	120.4	120.3	120.3	120.3
C12–C11–H18	109.2	108.8	108.8	108.8	108.8
C6–C12–C11	130.7	130.8	130.9	130.9	130.9
**Dihedral angle (º)**					
H13–C1–C2–C3	-180.0	-180.0	-180.0	-180.0	-179.99
H13–C1–C2–H14	0.0	0.0	0.0	0.0	0.0
N21–C1–C2–C3	0.0	0.0	0.0	0.0	0.0
N21–C1–C2–H14	180.0	180.0	180.0	180.0	179.9
C2–C1–N21–C5	0.0	0.0	0.0	0.0	0.0
H13–C1–N21–C5	-180.0	-180.0	-180.0	-180.0	-179.9
C1–C2–C3–C4	0.0	0.0	0.0	0.0	0.0
C1–C2–C3–C7	180.0	180.0	180.0	180.0	179.9
H14–C2–C3–C4	180.0	180.0	180.0	180.0	179.9
H14–C2–C3–C7	0.0	0.0	0.0	0.0	0.0
C2–C3–C4–C5	0.0	0.0	0.0	0.0	0.0
C2–C3–C4–N20	-180.0	-180.0	-180.0	-180.0	-179.9
C7–C3–C4–C5	-180.0	-180.0	-180.0	-180.0	-179.9
C7–C3–C4–N20	0.0	0.0	0.0	0.0	0.0
C2–C3–C7–C6	-180.0	-180.0	-180.0	-180.0	-179.9
C2–C3–C7–C8	0.0	0.0	0.0	0.0	0.0
C4–C3–C7–C6	0.0	0.0	0.0	0.0	0.0
C4–C3–C7–C8	180.0	180.0	180.0	180.0	179.9
C3–C4–C5–H15	180.0	180.0	180.0	180.0	179.9
C3–C4–C5–N21	0.0	0.0	0.0	0.0	0.0
N20–C4–C5–H15	0.0	0.0	0.0	0.0	-0.0
N20–C4–C5–N21	180.0	180.0	180.0	180.0	179.9
C3–C4–N20–C6	0.0	0.0	0.0	0.0	-0.0
C3–C4–N20–H16	-180.1	-180.0	-180.0	-180.0	-179.8
C5–C4–N20–C6	180.0	180.0	180.0	180.0	179.9
C5–C4–N20–H16	-0.1	0.0	0.0	0.0	0.1
C4–C5–N21–C1	0.0	0.0	0.0	0.0	0.0
H15–C5–N21–C1	180.0	180.0	180.0	180.0	179.9
C12–C6–C7–C3	180.0	180.0	180.0	180.0	179.9
C12–C6–C7–C8	0.0	0.0	0.0	0.0	-0

### 4.2 Mulliken charge orientation analysis

**Fig. 2:**
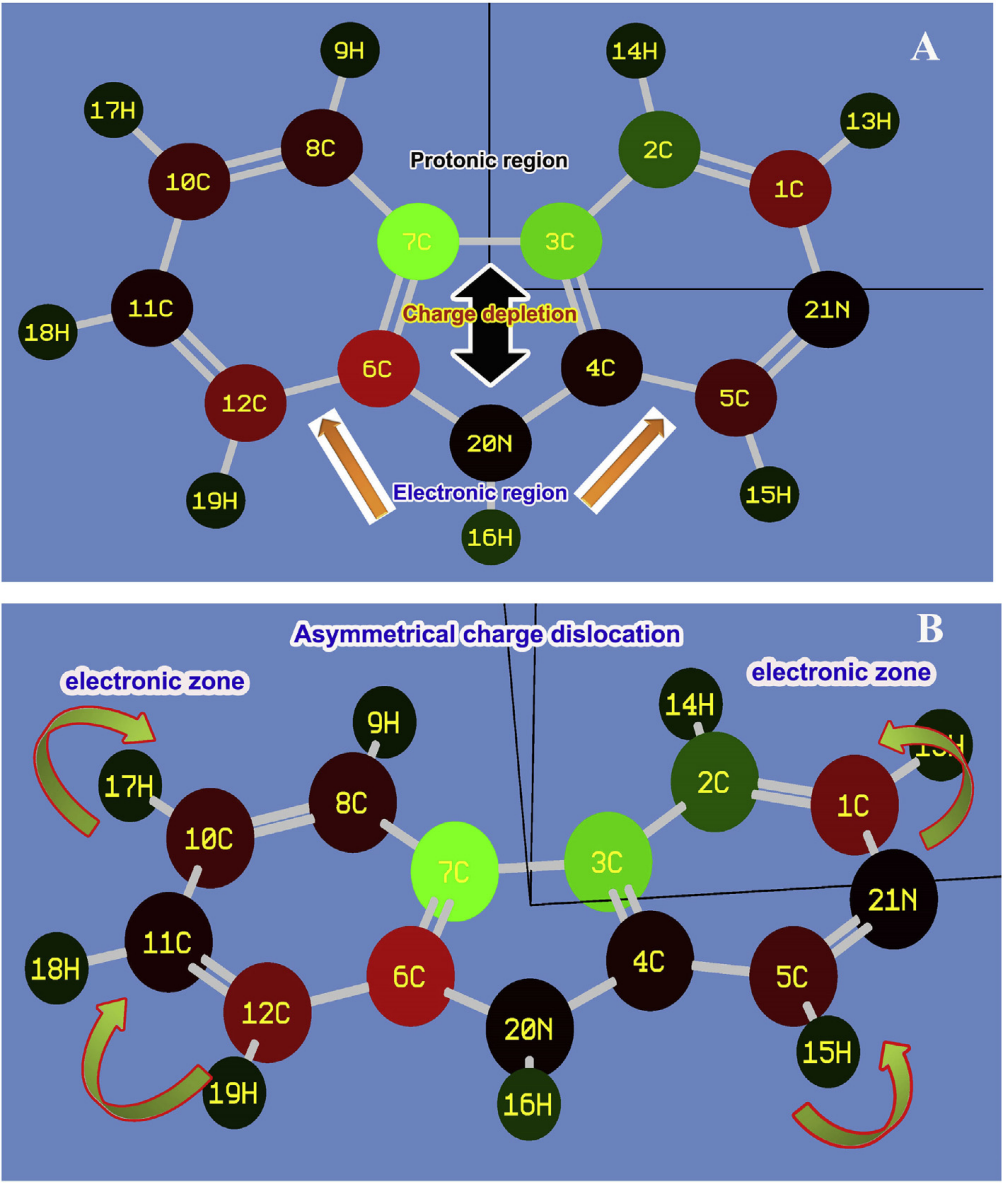
(A) vertical (B) horizontal view of mulliken charge assignment of Beta Carboline.

The molecular charge depletion process is used to identify the charge accumulation of various entities due to the orientation of chemical potential root. Usually, the charge depletion showed the presence of asymmetric charge domain which is exclusively explored the cause of drug potential in the chemical compound [19]. The charge modification was clearly showed in Fig. 2. Due to the Mulliken charge gradient, protonic content was appeared on top moiety of entire frame whereas the electronic content was moved on the bottom moiety of the frame. The strong dipoles were viewed on C5–N21 and C1–N21 in the pyridine segment and C6–N20 in pyrrole ring. Even though, CC on the benzene ring, another strong dipoles were present at C6–C7 and C7–C8 which were purposively induced consistent antibiotic activity in the compound.

The neutral atoms were present at C11, C21, N20 and N21 in which the chemical potential started their parametric oscillations over the molecule. These are the nodal region of the molecule and also the origin of the chemical equivalence potential (chemic-potential). The weak bonds were present at C2–C3 and C3–C7 on which the electronic content was evacuated. This was mainly due to the asymmetrical movement of the electronic domain to the needed portion of the molecule to induce required drug property. The remaining part of the core atoms were occupied by rather electronic content which were acting as core pathway for the flow of oscillating electronic potential.

### 4.3 Structure activity/Biological property analysis

**Fig. 3:**
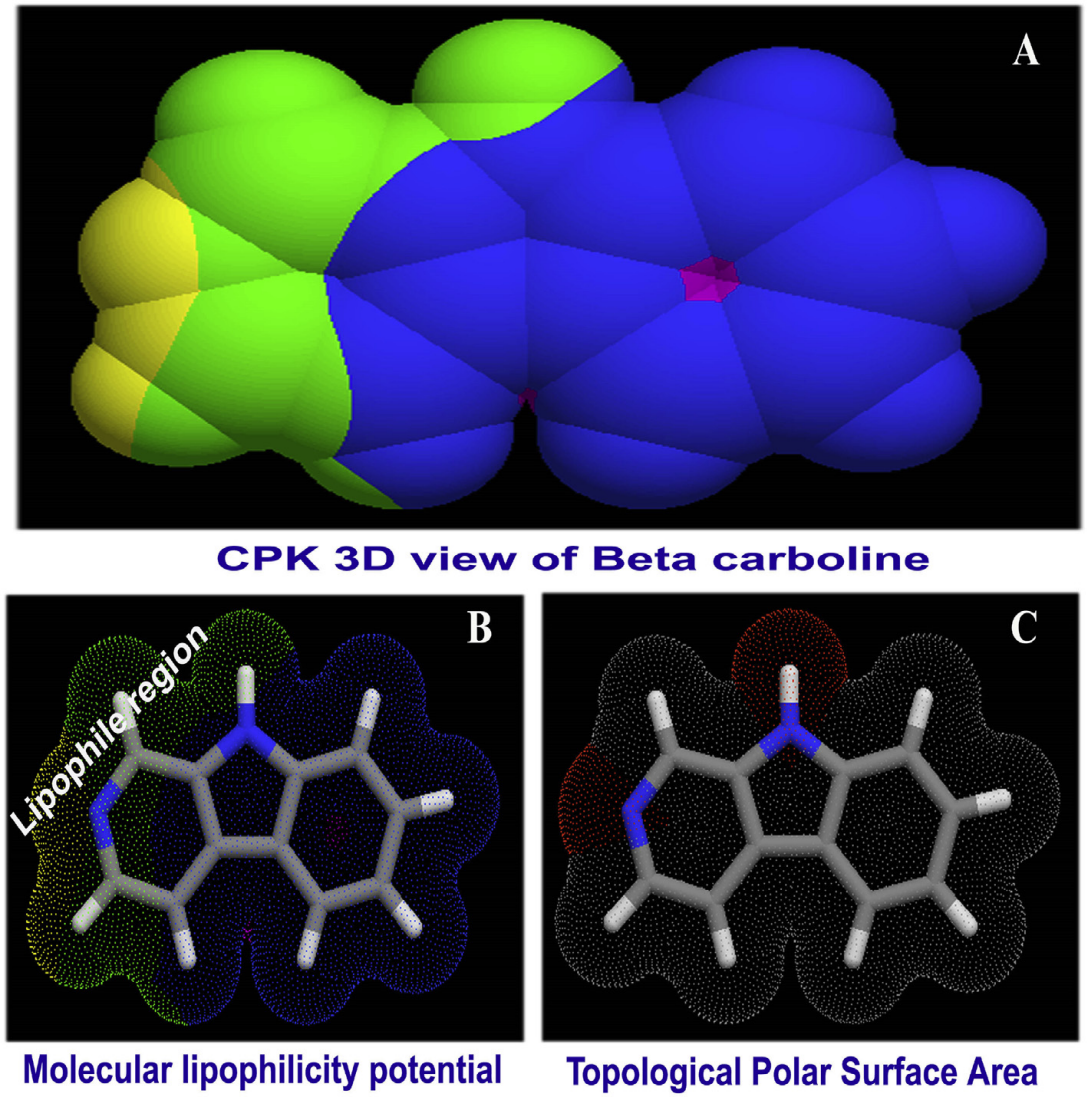
(A) CPK view (B) MLP view (C) TPSA view of Beta Carboline.

The Lipinski's rule of five (RO5) is a drug active selection rule of thumb which is used to estimate the druglikeness. Usually, it determines the pharmacological and biological activity of organic chemical compound due to which orally energetic drug for curing disease of human [19]. According to the Lipinski's rule, an orally active drug has not more than 5 hydrogen bond donors, not more than 10 hydrogen bond acceptors, molecular mass less than 500 Da, an octanol-water partition coefficient clog P not greater than 5.6, number of atoms within the range of 20–70 and rotatable bond count is less than 5. In this case, as in Table 2, HBD count, HBA count, topological surface area, Log P, n ON and Rotatable bond count were found to be in order; 1, 1, 168.0 g/mol, 2.37, 2 and 0 respectively. All such five parameters have been observed within the expected and allowed limit that refined the drug quality of the present compound; β-Carboline. The biological parametric diagrams are present in Fig. 3.

**Table 2:** Structure activity/Biological parameters of β-Carboline.

Parameters	Values
Hydrogen bond donor count	1
Hydrogen bond acceptor count	1
Rotatable bond count	0
Topological Polar Surface Area	28.7A^2^
Mono isotopic Mass	168.06 g/mol
Heavy Atom Count	13
Covalently-Bonded Unit Count	1
cLogP	2.37 no unit
n atoms	13
MW	168.20 g/mol.
n ON	2
n OHNH	1
n violations	0
n rotb	0
volume	152.86 μ^3^
GPCR ligand	0.11
Ion channel modulator	0.66
Kinase inhibitor	0.38 mg/mL
Nuclear receptor ligand	0.75
Protease inhibitor	0.57 mg/mL
Enzyme inhibitor	0.23 mg/mL

The G protein-coupled receptors are also known as G protein-linked receptors which sense of detect organic drug molecules arrived outside the cell that activate inside signal transduction pathways and cellular responses. Here, the value of such parameter was determined to be 0.11 which was able to activate an associated G-protein by exchanging its bound GDP for a GTP. The present drug was found to be able to pass through the cell membrane. The ion channel modulator is an ability of drugs which modulate the ion channels and they include channel blocker and channel opener. Here, the coefficient of modulator was calculated to be 0.66 which was moderate and the present the compound was having capability and biological mechanism to modulate the associated signal to the drug target.

The Kinase inhibitor of the present compound was observed to be 0.38, was rather reasonable that inhibit specific kinases is being developed to treat the disease. This inhibition value of the present compound was enough that specifically blocks the action of protein kinases. The Nuclear receptor ligand of the title compound was to be 0.75 which was high to have the ability to directly bind to protein interact with and control and regulate the expression of adjacent genes. The Protease inhibitor play prime role in life cycle of microbes, hindering the action of proteases which was found to be 0.57 for of the present case. This was supposed to be specific in nature as they target and block only the proteases and other proteins stay unaffected. All the enzymatic process for the biological active substance like present case was well agreed with the literature [20]. The present chemical substance was able to potent inhibition of the development of an extensive array of pathogenic bacteria. The Enzyme inhibitor coefficient was appeared to be 0.23 and even though it was very low, it was capable of cessation in the enzyme activity. From the above observation, it was concluded that, the present drug compound having enriched absorption, distribution, metabolism and Excretion with high efficacy.

### 4.4 Vibrational investigation

#### 4.4.1 Vibrational assignments

**Fig. 4:**
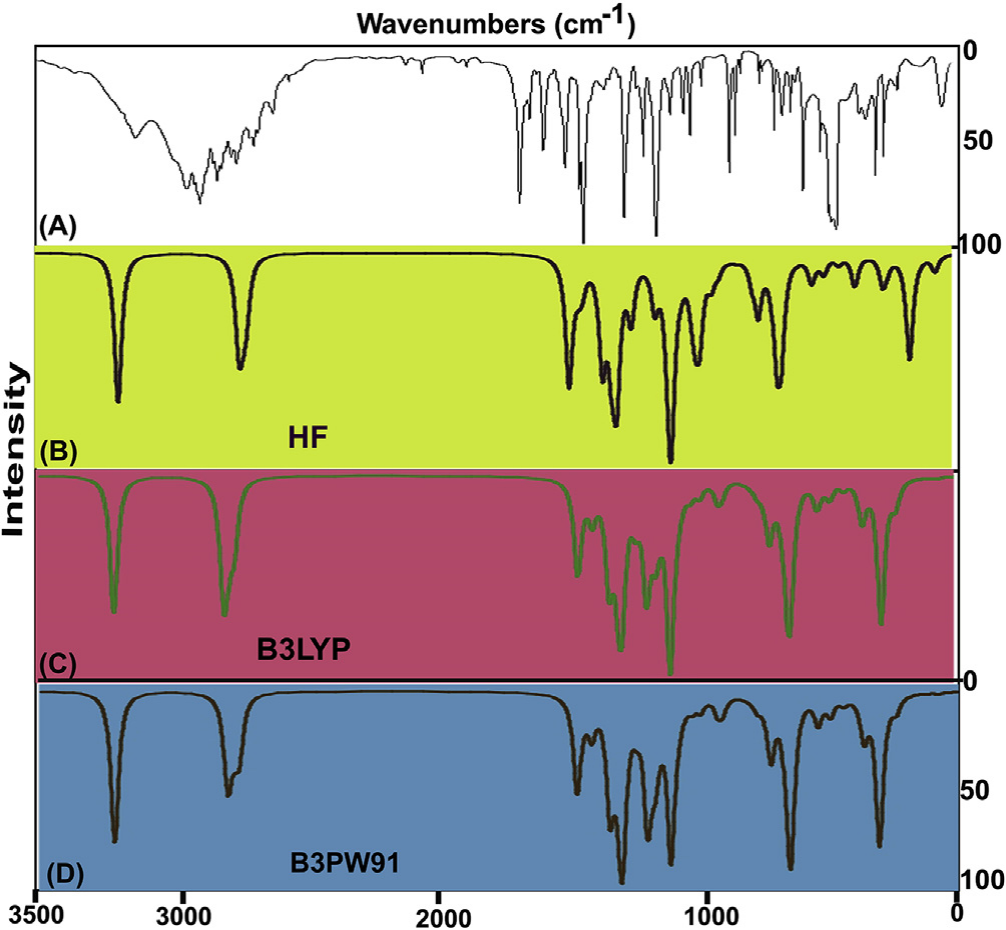
(A) Experimental and Calculated FT-IR spectra (B) HF (C) B3LYP (D) B3PW91 of Beta Carboline.

**Fig. 5:**
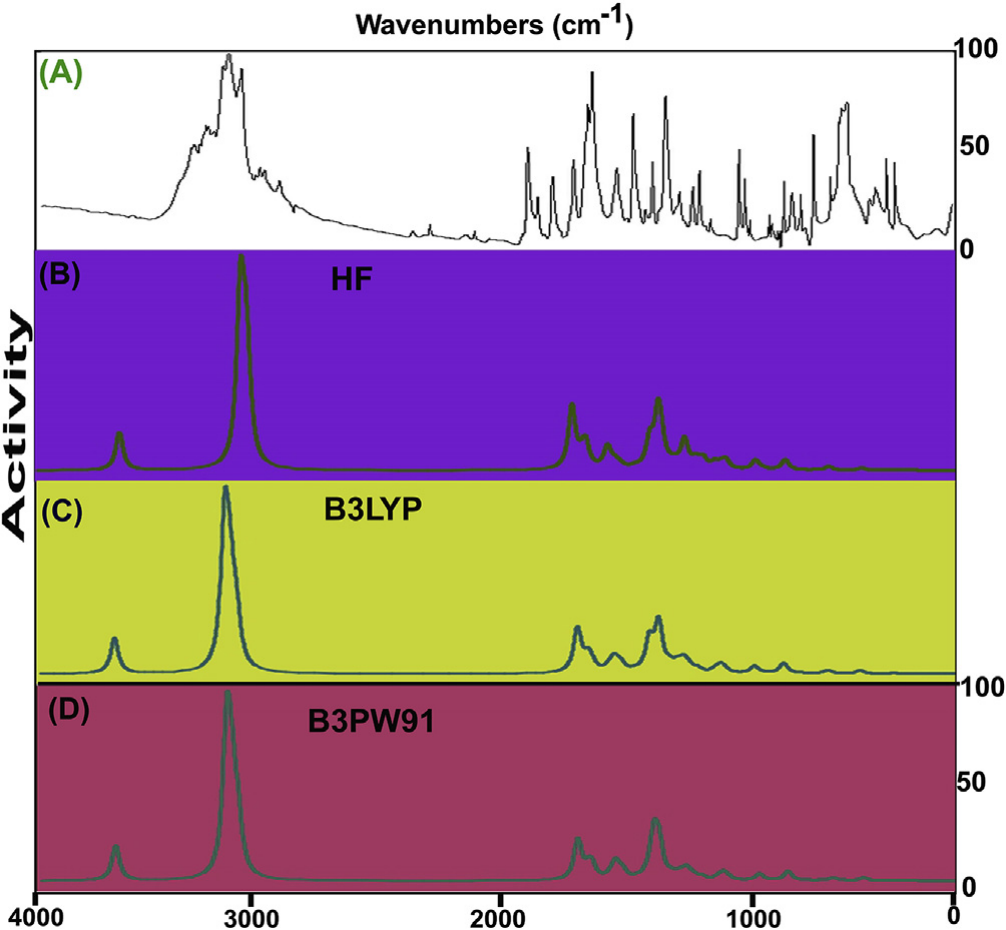
(A) Experimental and Calculated FT-Raman spectra (B) HF (C) B3LYP (D) B3PW91 of Beta Carboline.

The FT-IR and FT-Raman vibrational frequency pattern was presented in Table 3 and experimented and work out spectra using HF and DFT theories was demonstrated in Figs. 4 and 5 respectively. There were 21atoms were present in the molecule that undergo vibrations were arranged with respect to their energy levels. As per the rules of irreducible depiction, the molecular structure of this case comes under the *C*_S_ point group and 63 vibrational bands were found. Such 57 compositional fundamental modes were systematically divided as; 21 stretching modes, 18 in plane bending vibrations and 18 out of plane bending vibrations. The finger print and group frequencies with strong to weak intensity were observed with respect to their characteristics motion in the region 50- 3600 cm^−1^. As per the selection rule, the Aʹ and Aʺ species were allocated for related modes of vibrations.

Γ_63_Vibrations = 39Aʹ+ 18Aʺ

**Table 3:** Observed and calculated vibrational frequencies of Beta Carboline.

Symmetry Species C_s_	Observed Frequency(cm-^1^)		Methods					Vibrational Assignments
			HF	B3LYP	B3LYP	B3PW91	B3PW91	
	FT-IR	FT-Raman	6-311++ G(d, p)	6-31++G (d, p)	6-311++G (d, p)	6-31++G (d, p)	6-311++G (d, p)	
A**′**	3420vs	-	3411	3514	3515	3527	3527	(N–H) **υ**
A**′**	3070w	-	3109	3064	3059	3067	3062	(C–H) **υ**
A**′**	-	3060vs	3108	3056	3052	3062	3056	(C–H) **υ**
A**′**	-	3040vs	3098	3053	3048	3057	3053	(C–H) **υ**
A**′**	-	3010vs	3088	3044	3039	3048	3043	(C–H) **υ**
A**′**	-	2970vs	2900	3037	3033	3042	3036	(C–H) **υ**
A**′**	2960m	-	2894	3035	3026	3035	3027	(C–H) **υ**
A**′**	-	2940s	2887	2727	2722	2760	2721	(C–H) **υ**
A**′**	1600s	1600m	1577	1595	1591	1603	1603	(C=N) **υ**
A**′**	1595s	-	1556	1592	1591	1603	1580	(C=C) **υ**
A**′**	1580vs	-	1535	1569	1568	1579	1556	(C=C) **υ**
A**′**	1560s	1560s	1532	1547	1546	1556	1542	(C=C) **υ**
A**′**	1550s	-	1523	1531	1529	1542	1600	(C=C) **υ**
A**′**	1495s	-	1520	1440	1439	1445	1444	(C=C) **υ**
A**′**	1465m	1465vs	1492	1425	1423	1426	1425	(C=C) **υ**
A**′**	1390vs	-	1382	1409	1408	1414	1413	(C–N) **υ**
A**′**	1300vs	-	1323	1353	1351	1359	1356	(C–N) **υ**
A**′**	1280vs	1280s	1260	1307	1302	1314	1310	(C–N) **υ**
A**′**	1205vs	-	1228	1271	1263	1213	1213	(N–H) δ
A**′**	1200vs	-	1213	1208	1207	1189	1189	(C–C) **υ**
A**′**	-	1175m	1177	1187	1187	1185	1176	(C–C) **υ**
A**′**	1170vs	1170m	1163	1174	1174	1176	1169	(C–C) **υ**
A**′**	1150vs	-	1154	1167	1169	1175	1156	(C–C) **υ**
A**′**	1145vs	-	1144	1147	1149	1157	1143	(C–C) **υ**
A**′**	-	1120vw	1127	1136	1133	1150	1152	(C–C) **υ**
A**′**	1110vs	-	1119	1124	1127	1121	1123	(C–H) δ
A**′**	1040s	1040s	1061	1087	1089	1087	1089	(C–H) δ
A**′**	1020vs	-	1043	1014	1016	1017	1018	(C–H) δ
A**′**	-	1010vw	1031	995	996	997	998	(C–H) δ
A**′**	960w	-	1017	978	982	977	981	(C–H) δ
A**′**	950vs	950vs	951	939	938	937	934	(C–H) δ
A**′**	945vs	-	949	926	928	924	926	(C–H) δ
A**″**	905vs	-	931	905	905	902	904	(N–H) γ
A**″**	890vs	-	893	876	898	874	871	(C–H) γ
A**″**	870vs	870m	886	850	894	848	853	(C–H) γ
A**″**	865vs	-	885	899	874	903	903	(C–H) γ
A**″**	850vs	-	858	897	869	897	893	(C–H) γ
A**″**	845vs	-	851	870	854	870	870	(C–H) γ
A**″**	-	830vs	801	821	819	818	818	(C–H) γ
A**″**	780vs	-	775	795	798	797	799	(C–H) γ
A**′**	760vw	-	757	792	791	791	790	(CNC) δ
A**′**	755vw	755vs	756	776	777	776	778	(CCC) δ
A**′**	680vs	-	747	713	716	712	714	(CCC) δ
A**′**	650vs	-	689	657	660	654	656	(CCC) δ
A**′**	610s	-	618	585	586	584	585	(CCC) δ
A**′**	595vs	-	578	602	601	602	601	(CCC) δ
A**′**	560vs	-	567	544	548	541	545	(CNC) δ
A**′**	445w	-	479	446	449	450	447	(C–C) δ
A**″**	-	410w	429	427	425	426	424	(CNC) γ
A**″**	-	360w	350	375	376	378	375	(CCC) γ
A**″**	-	330w	338	322	324	326	321	(CCC) γ
A**″**	-	290w	298	303	301	274	312	(CCC) γ
A**″**	-	280w	280	280	280	275	280	(CCC) γ
A**″**	-	220w	218	213	211	218	214	(CCC) γ
A**″**	-	160w	161	165	166	167	166	(CNC) γ
A**″**	-	110w	110	113	113	114	113	(C–C) γ
A**″**	-	100w	100	101	100	101	100	(N–H) τ

#### 4.4.2 Indole core C=C and C–C vibrations

Typically, in indole ring derivatives, the carbon base core ring reveals its core frame vibrations such as C=C and C–C stretching modes in the region 1390-1580 cm^−1^ [21]. Accordingly, here, the core carbon bonds were conquered and its C=C stretching bands were observed with very strong absorption coefficient at 1595, 1580, 1560, 1550, 1495 and 1465 cm^−1^ whereas the C–C stretching bands have been found with strong to medium intensity at 1200, 1175, 1170, 1150, 1145 and 1120 cm^−1^. All the π-bonding stretching vibrations were found above and well within the limit of the characteristics region though indole was adopted with pyridine ring. Here, these vibrations including pyridine carbon core vibrations and all the vibrational modes resonance with one another and perfectly merged with one another. All the stretching associated with σ-associated CC bonds were purposively moved down to well below the lower limit of the allotted region. Here, the σ-bonds found alternatively between π-bonds and from which it was clear that, the σ-interaction bond energy was utilized to arrange such drug activity in the compound. Supportively, the CCC in plane and out of plane bending modes were observed at 755, 680, 650, 610 and 595 cm^−1^ and 360, 330, 290, 280 and 220 cm^−1^ respectively. All the above bending signals showed the consumption of low vibrational energy by the added core segments.

#### 4.4.3 Core C=N and C–N vibrations

The core imine group vibrational stretching is found in the region of 1580–1520 cm^−1^ for pyridine adopted ring vibrations [22]. In this case, the C=N stretching mode was appeared at 1600 cm^−1^. Form the observed value; it was elevated up well above the characteristics region and it is always found top end or above the expected limit due to this the imine group was proved its stability. If the imine group stabilized well in the molecule, the vibrational impression will be very much and instantly, the drug property was enhanced.

In this case, the C–N bond was appeared in two places; such as, two at pyrrole ring and one was found at pyridine ring. According to the mutual exclusion principle, for core C–N is usually observed in the region 1350-1250 cm^−1^ [23]. Hence, in the present case, the stretching bands were taking place at 1390, 1300 and 1280 cm^−1^in IR only. Wherever the bond, the related stretching modes were observed above and within the expected region of the spectrum and it also proved its strong dipole moment character by appearing on IR only. Correspondingly, the allied CNC in plane and out of plane breathing modes were seen at 760 & 560 cm^−1^ and 410 & 160 cm^−1^ respectively. In the vibrational power of the heteronuclear bond, there was no alternation of vibrational energy found to develop the related property of the compound.

#### 4.4.4 N–H vibrational pattern

In general, in the case of indole derivative, the harmonic oscillation regarding to N–H stretching is observed in the region 3480-3160 cm^−1^ [24, 25] while consuming IR radiations. Relatively, in the case of indole derivatives, usually, the N–H scissoring and wagging vibrational frequencies are appeared in the region 1460-1320 cm^−1^ and 880-700 cm^−1^ respectively [26]. In the present molecule, the N–H stretching, scissoring and wagging bending modes have been identified at 3420, 1205 and 905 cm^−1^ correspondingly. As stated by the previous work [27], except N–H scissoring signal, the appeared bands of stretching and wagging modes was observed precisely on the expected region which symbolized the amino group strength in the compound. Routinely, N–H vibrations have not affected by linked or unrelated chemical species. But in this case, the indole was coupled with pyridine ring by which the part of energy was consumed by C–N on both side of rings (low energy for bending vibrations) and due to this reason, the in plane bending; scissoring mode was rather suppressed. This vibrational consumption process was taking place in the molecule enhance the involvement of N–H and C–N bonds in the making drug intensiveness.

#### 4.4.5 C–H vibrational energy progression

As regards the C–H vibrations in the case of indole derivatives, the C–H stretching vibrations for benzene and pyridine rings are predicted to present in the region 3100-3000 cm^−1^ and 3100-3010 cm^−1^ [28, 29] respectively. As a result, in this chemical species, the benzene, pyridine and pyrrole ring were fused together and formed the β-Carboline compound in which, the C–H was located in three different rings. Here, the C–H stretching wavenumbers were observed at 3070, 3060, 3040 and 3010 cm^−1^ in Raman spectrum for benzene ring whereas the vibrational bands were found at 2970, 2960 and 2940 cm^−1^ in IR and Raman for pyridine ring. Since the benzene ring was in the indole base whereas the pyridine was the substitutional ring, the C–H allied bond stretching bands were severely infected. This was mainly due to the pyridine ring was devoted the required potential to the indole ring for initiating and stabilizing antibiotic drug activity.

However, the C–H in plane and out of plane bending vibrations are commonly found in the region 1300 - 1010 cm^−1^ and 950–700 cm^−1^ respectively [30]. Here, in plane and out of plane bending bands were found at 1110, 1040, 1020 and 1010 cm^−1^ and 890, 870, 865 and 850 cm^−1^ respectively for benzene ring. In the case of pyridine ring, the in plane and out of plane bending signals were determined to be located at 960, 950 and 945 cm^−1^ and 845, 830 and 780 cm^−1^ respectively. Similar to the stretching bands, the entire bending modes for benzene ring was observed within the expected region whereas all the bending modes for pyridine ring was miserable greatly which was due to the mid IR region energy equivalent chemical potential was utilized to fabricate the biological activity in the present compound.

### 4.5 NMR exploration

The NMR spectral analysis provide the information regarding the arrangement of chemical reaction path to estimate of rate of drug gain over the molecule by the injection of passive and active substitutional group with the strong fixed base structure [31]. It also make available chemical modulation rate on core carbon atoms by the supplementary effect of electron withdrawing and donor substitutional groups in the base molecule [32]. The injection of ligand groups in the specific point on the standard molecular structure is usually measured by the appropriate chemical shift [33].

**Fig. 6:**
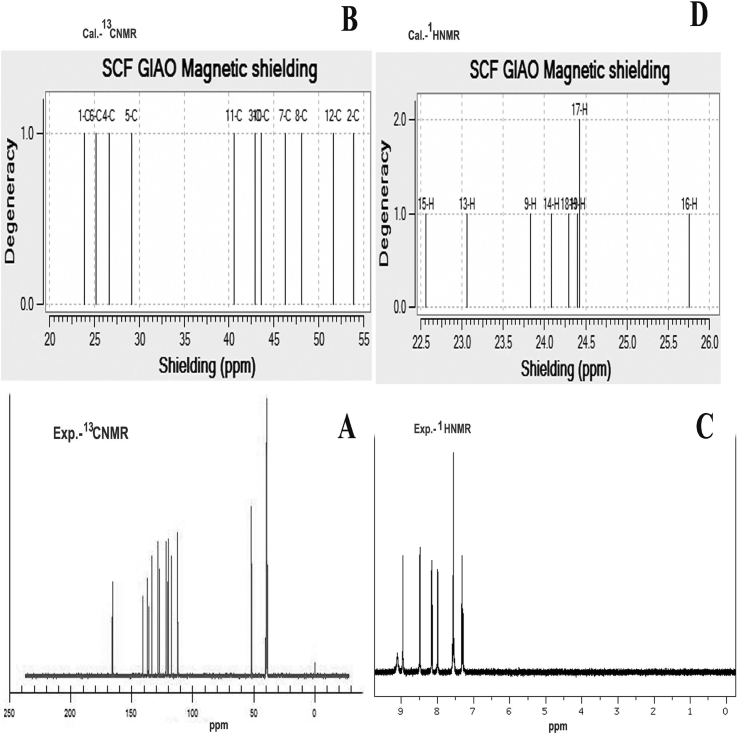
Experimental ^13^C NMR (A) ^1^H NMR (C) and Calculated ^13^C NMR (B) ^1^H NMR (D) spectra of Beta Carboline.

The NMR spectra for this molecular composite are showed in Fig. 6 and the measured value of the chemical shift was clearly displayed in Table 4. At this juncture, as usual, the chemical shift of C1 and C5 at the point of heteronuclear bond was found to be 158(expt. 166 ppm) and 153 (expt. 165 ppm) respectively which was very high when compared with other core atoms. Similarly, the chemical shift of C4 and C6 were determined to be 155 (expt. 165 ppm) and 157 ppm (165 ppm) respectively which was another location in which the heteronuclear bond was present. In this place, the chemical shift was observed to be very high due to the random deshielding effect by active electron sucking character of N. The chemical shift of C11 and C3 were identified to be 141 and 139 ppm respectively which were also high and this was mainly by the sweep up of electrons from the specific shield around the carbons in the benzene ring. These electrons were formed in the form of cloud to restore the negative potential in the frontier of the imine group. The chemical shift of further core parts of the molecule was ranged from 130-138 ppm where in which no heteronuclear bond was appeared. From the chemical shift of entire core carbons, it was observed that, the chemical reaction potential was originated at C–N and C=N over the molecular system. The chemical path way was started from N20–C4–C5–N21 and was regulated to C1 and C6. This view of reaction path mechanism streamlined chemical equivalent potential which was produced on the imine group and it was stabilized by other C–N bonds in the molecule. So the compound would be an effective antibiotic drug being with anticancer activity. The experimental chemical shit was observed for DMSO solvent only whereas in the case of theoretical, the chemical shift was observed in DMSO and CCl_4_ solvents. The solvent effect was rather observed in experimental and theoretical results.

**Table 4:** Experimental and calculated ^1^H and^13^C NMR chemical shifts (ppm) of Beta Carboline.

Atom position	TMS-B3LYP/6-311++G (2d,p) Shift (ppm)			Experimental shift (ppm)
	Gas	Solvent phase		
		DMSO	CCl_4_	
C1	158.6	157.5	158.2	166
C2	123.8	130.6	129.4	128
C3	139.6	140.1	139.8	136
C4	155.8	156.6	156.1	165
C5	153.3	155.8	154.4	-
C6	157.3	158.2	157.7	165
C7	136.2	135.4	135.9	134
C8	134.4	134.8	134.5	136
C10	138.9	139.4	139.1	138
C11	141.9	142.8	142.3	142
C12	130.8	131.8	131.2	124
H9	8.8	8.3	8.1	8.2
H13	9.5	8.8	8.8	8.8
H14	8.5	8.1	7.9	8.1
H15	10.0	9.4	9.4	9.3
H16	6.8	6.8	6.4	-
H17	24.4	7.6	7.5	7.6
H18	8.3	7.8	7.7	7.9
H19	8.2	7.8	7.6	-

### 4.6 FMO interaction examination

**Fig. 7:**
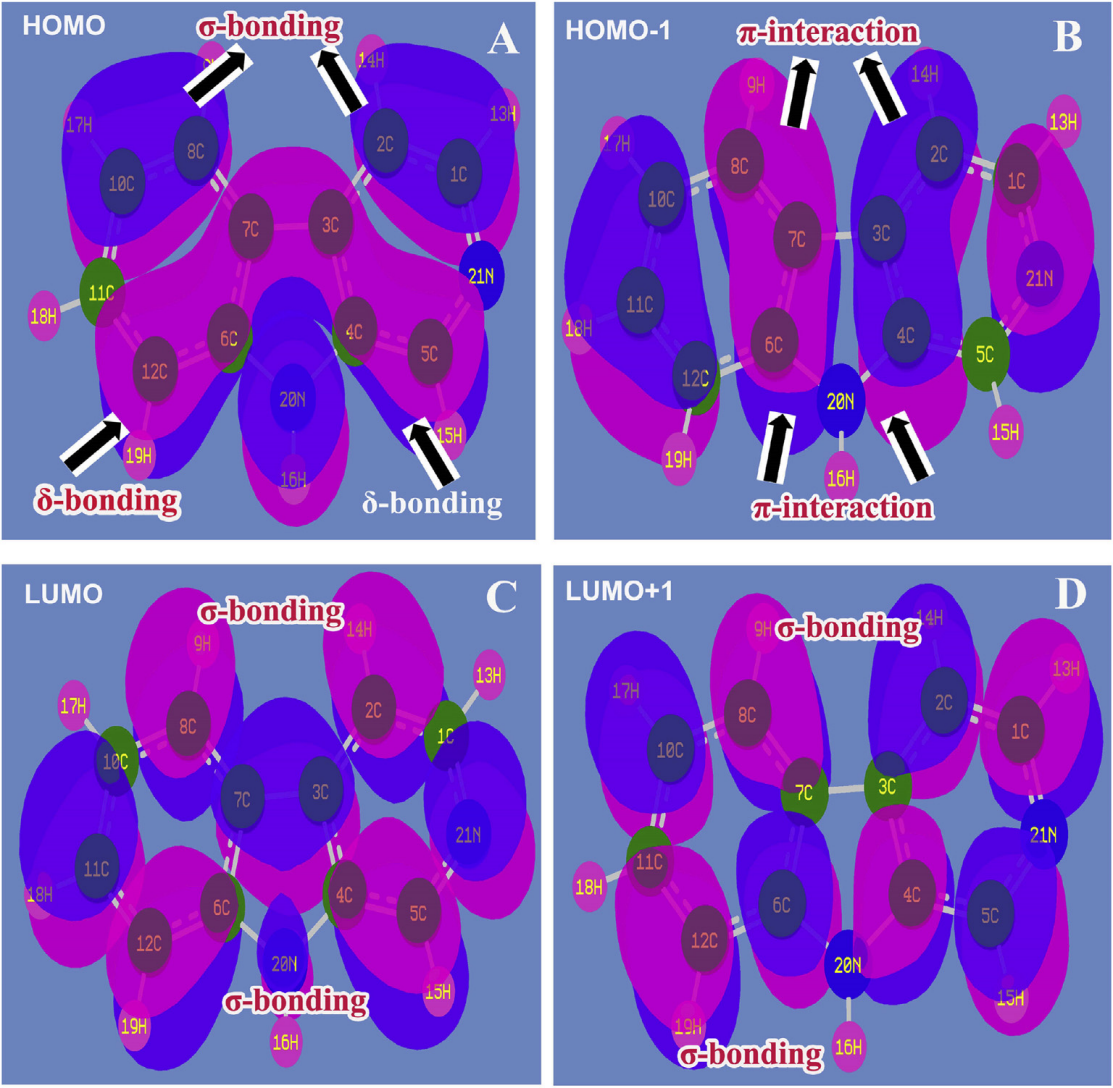
Frontier (A) HOMO (B) HOMO-1 (C) LUMO (D) LUMO+1 of Beta Carboline.

The frontier molecular orbital (FMO) interaction usually taking place between HOMO and LUMO sequence in which the important interaction profile explained the details of π, σ and δ-bonding system interaction over the different entities of the molecule which leads the cause of chemical property and biological potency [34]. Usually, the π-interaction overlapping system belongs to the molecule promotes the drug effectiveness and therapeutic efficiency. The chemical energy transformation from base to ligand entity or ligand to base core is determining the overall response to the drug. The chemical reactivity for drug responses can be accurately predicted by organizing orbital interactions in different parts of the molecule. The entire interaction system was presented in Fig. 7 and the transitional values were found in Table 5.

**Table 5:** Frontier molecular orbitals of Beta Carboline with energy levels.

Energy levels	Frequency region B3LYP/6-311++G(d,p) (eV)	UV-Visible region (eV)
H+10	11.31	11.19
H+9	10.55	10.76
H+8	10.52	10.68
H+7	10.00	10.02
H+6	9.74	9.62
H+5	9.59	9.60
H+4	8.47	8.46
H+3	7.40	7.51
H+2	7.11	7.22
H+1	6.63	6.76
H	6.10	6.09
L	1.57	1.41
L-1	0.47	0.43
L-2	0.43	0.16
L-3	0.13	0.00
L-4	0.04	0.12
L-5	0.06	0.17
L-6	0.31	0.26
L-7	0.45	0.61
L-8	0.57	0.68
L-9	0.93	1.01
L-10	1.05	1.16

In LUMO, though, there were number of σ-interaction orbital overlapping appeared in the form of vacant interactive-orbital lobes and these were ready to receive the electronic energy to create the drug potency. In HOMO, the δ-bonding produced the interaction of orbitals in which the excited electron cloud was found to be donating the electronic energy to the lowest unoccupied orbital contour to cook the antibiotic property. The pyrrole carbons; C5, C4, C3, C7, C6 and C12 were directly involved in the energy transitional process in terms of δ-interacting system and the chemi-potential energy was transferred from δ-overlapping space orbital system to σ-orbital interactive interface orbital system. This exchange of electronic orbital chemi-kinetic energies between the HOMO and LUMO sequence, generate hybrid form of potential and it was further induced profound antibiotic energy. In this case, first order orbitals interaction takes place for the amendment of such drug property. Similarly, in the second order LUMO interacting system, the σ-interaction orbital overlapping was appeared whereas in second order HOMO profile, there were number of π-interaction space orbital interaction found which covered the semicircle carbon core system like; C10, C11, C12; C6, C7, C8; C2, C3, C4; C1,N21. This π-interacting system enabled the electron cloud donation as a series transitions to σ-interaction vacant orbital system. Such sequential form of exchange of electronic chemi-potential formed successful antibiotic property.

### 4.7 CT complex identification

**Fig. 8:**
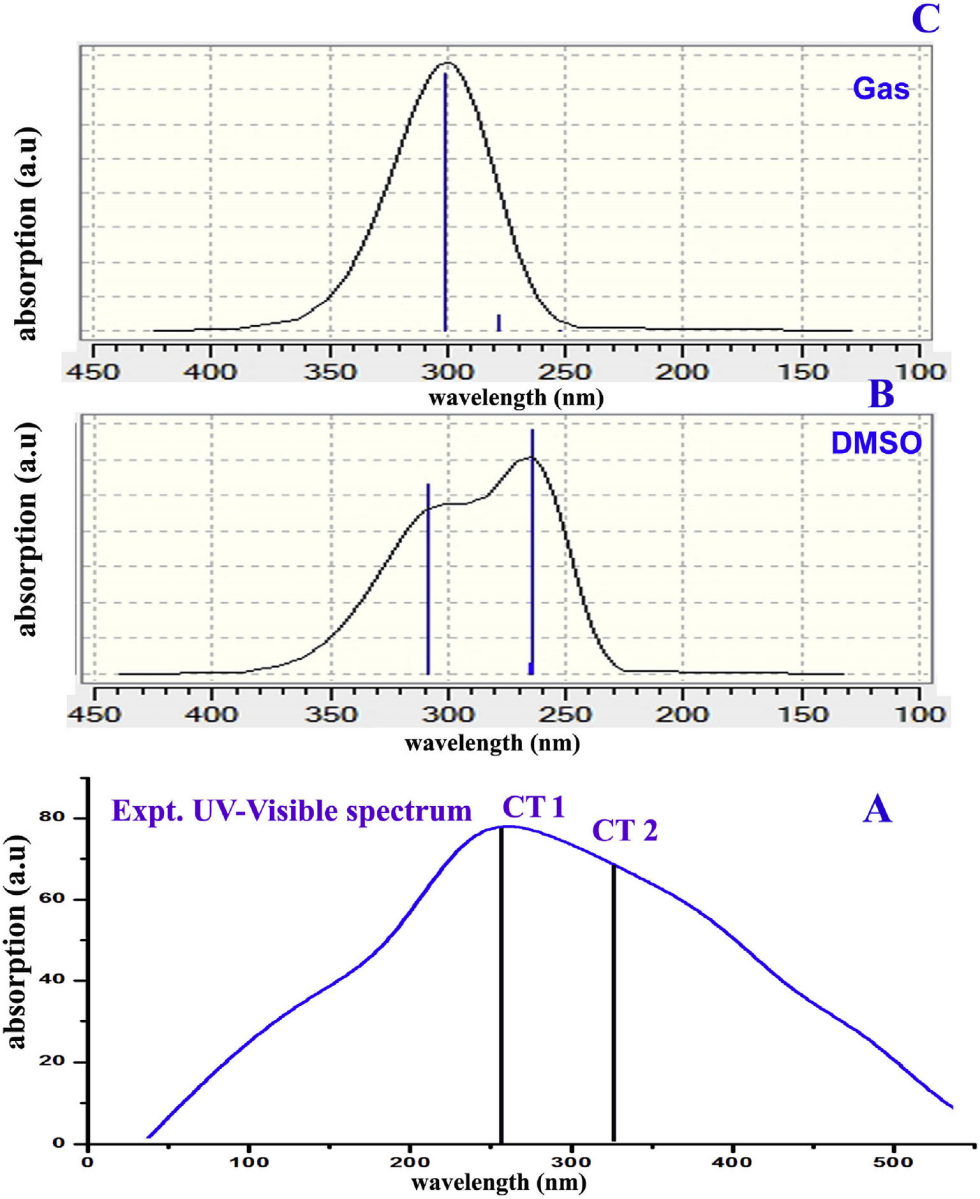
(A) Experimental (B) & (C) Calculated UV-Visible spectrum of Beta Carboline.

The calculated and experimental UV-Visible absorption spectra were put on view in Fig. 8 and the respected determined values are depicted in Table 6. The absorption spectra were completely drawn from the electronic transitional pattern which was in receipt of the collection of vibrational transitional model. The absorptional spectrum represented the formation of charge transfer complex which is the major key part to generate the drug characteristics of the chemical species. The spectral peak is usually shifted by the application of configure of CT complex in the molecular system and here, two separate peaks were identified in the experimental as well as theoretical spectrum such that at 260 nm & 330 nm and 265nm & 310 nm respectively.

**Table 6:** Theoretical electronic absorption spectra of Beta Carboline.

λ (nm)	E (eV)	(f)	Transition Level	Major contribution	Assignment	Region	Bands
**Gas**							
301.17	4.1168	0.0373	H→L (69%)	H→L (69%)	n→ σ *	Quartz UV	R-band (German, radikalartig)
278.71	4.4485	0.0023	H+2→L (68%)	H+2→L (68%)	n→π*		
252.09	4.9183	0.001	H→L-1 (70%)	H→L-1 (70%)	n→π*		
**DMSO**							
307.95	4.0261	0.0530	H→L (69%)	H→L (69%)	n→ σ *	Quartz UV	R-band (German, radikalartig)
265.17	4.6757	0.0031	H+2→L (69%)	H+2→L (69%)	n→π*		
263.70	4.7018	0.0685	H+1→L (59%) H→L-1 (37%)	H+1→L (59%)	n→π*		
**CCl**_**4**_							
304.92	4.0661	0.0549	H→L (69%)	H→L (69%)	n→ σ *	Quartz UV	R-band (German, radikalartig)
272.96	4.5422	0.0030	H+2→L (68%	H+2→L (68%)	n→π*		
262.89	4.7162	0.0883	H+1→L (60%)	H+1→L (60%)	n→π*		

H: HOMO; L: LUMO.

For the calculated spectrum, discrete transitions were observed at 301, 278 and 252 with the band gap of 4.11, 4.4 and 4.9 eV at oscillator strength of 0.03, 0.002 and 0.001 respectively. for DMSO and CCl_4_ solvent phase, the set of transitions were observed at 307, 265 and 263 with 4.02, 4.67 and 4.70 ev band gap and they were oscillated with 0.05, 0.003 and 0.068 respectively. Similarly, 304, 272 and 262 with band gap of 4.06, 4.54 and 4.71 ev which were oscillated by 0.05, 0.003 and 0.08 respectively. They were assigned to first order and second order HOMO and LUMO sequence and the transitions; n→ σ* and n→π* were allotted at R-Band in quartz region of the spectrum for gas state. The entire transitions in solvent phase were perfectly aligned with the gas phase. Here, two assigned electronic transitions; n→ σ* and n→π* were strongly confirmed the CT complex; C–N and C=N. here, one of the two CT complex was found at pyridine ring and another was assigned at pyrrole ring. Hence, it was well-known that, if the CT complex is imine group, the extraordinary antibiotic characteristics are produced in the related molecule. Here, the imine group along with heteronuclear bond as CT complex was recognized as good antibiotic agent. Both were recognized within the molecule that was ensured the rich biological activity.

### 4.8 MEP characterization

**Fig. 9:**
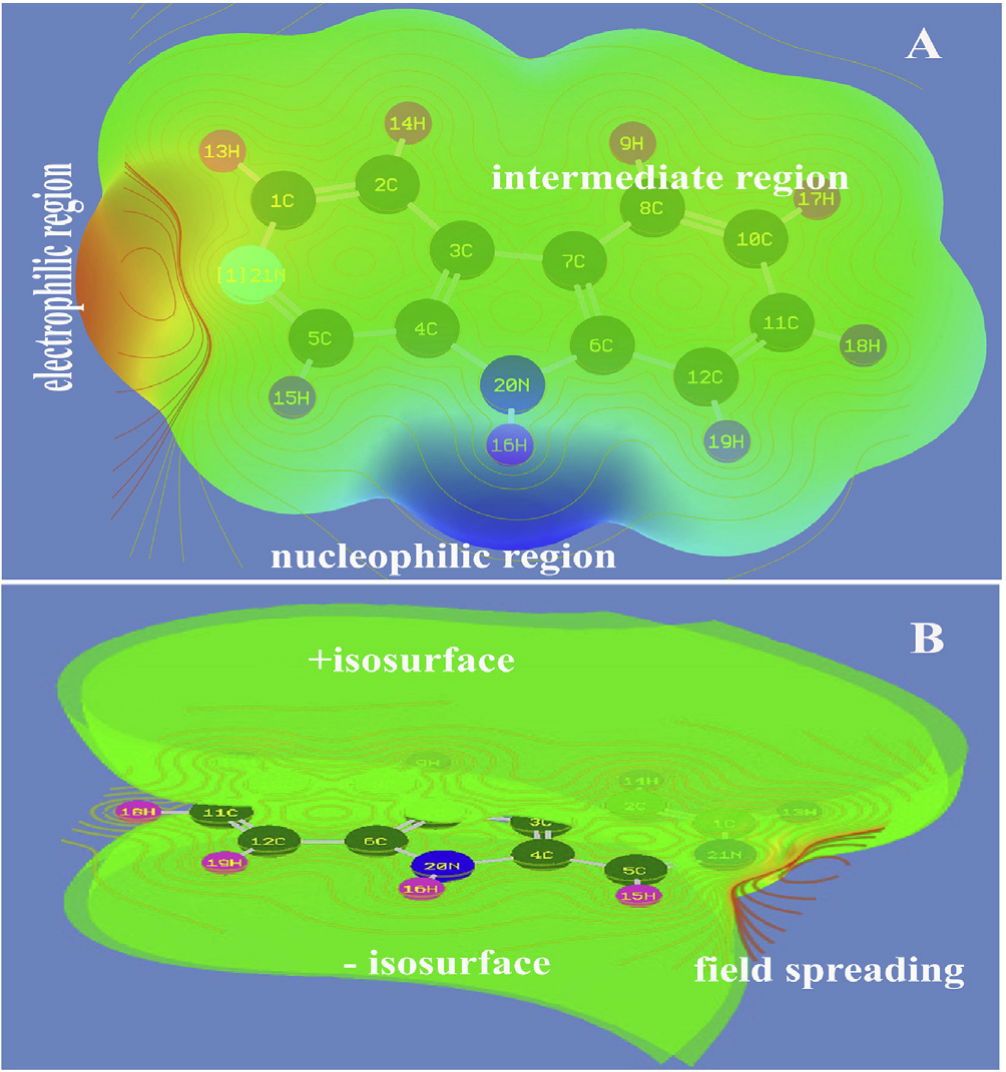
(A) MEP depletion view (B) MEP field zone view of Beta Carboline.

The molecular electrostatic potential is a map in which the detailed information regarding molecular reactivity and biological activity can be obtained [35]. The spatial field distribution around the molecule established electrophilic and nucleophilic entities which induced the appropriate chemical reaction and it is also responsible for the binding of a ligand at the active site of the receptor. The molecular electrostatic potential maps are usually used to find similar molecules which have coherent physical property and therefore locate essential features necessary for a certain biological activity [36, 37]. The Kohonen maps was indicated in Fig. 9 in which the influence of hetero atoms on the 3D molecular electrostatic potential (MEP) was represented.

In the present case, the negative zone called electrophilic region, appeared on the N of the pyridine ring where, it always attract the positive entities of the reactive molecule. Here, the electron acceptor reaction can be taking place on that point of the molecule and the point of electrophilic region at which the accepted reactive path can be linked to make contact with specified protein. The nucleophilic region was found on N–H bond of the pyrrole ring where the electron withdrawing group can be reacted and through which the entire chemical reactivity was processed and thus, the biological activity of the compound was recognized. Similarly, the potential field distribution over the space was appeared on the imine group; C=N and the N–H group that opened the potential to create the drug property.

### 4.9 Physico-chemical properties

As per Table 7, the zero point vibrational energy of the present case was found to be -533.63 and -553.46 Hartree for both IR and UV-visible region. As a drug agent, the value should be greater than 200 Hartree and the value of the present case was found beyond the expected limit which ensured the drug compound. The energy gap of the drug reactivity was determined to be 4.53 and 4.68 eV respectively in the IR and UV-Visible region. This value of energy gap was observed to be very high by which the consistent drug property could not be altered by the external reaction by chemical agent. The chemical hardness of the compound was 2.26 and 2.34 and both were traced to be moderate that powerfully indicated the present compound was being chemically strong and rigid.

**Table 7:** Calculated Physico-chemical parameters.

Parameter	B3LYP 6311++G (d,p)	UV-Visible	Electrophilicity charge transfer (E_CT_) (ΔN_max_)_A_-(ΔN_max_)_B_
E_total_ (Hartree)	-533.63	-533.49	
E_HOMO_ (eV)	6.102	6.08	
E_LUMO_ (eV)	1.56	1.40	
ΔE_HOMO-LUMO gap_ (eV)	4.53	4.68	
E_HOMO-1_ (eV)	6.62	6.75	-0.309
E_LUMO+1_ (eV)	0.46	0.43	
ΔE_HOMO-1-LUMO+1 gap_ (eV)	6.16	6.32	
Chemical hardness (η)	2.26	2.34	
Electronegativity (χ)	3.83	3.74	
Chemical potential (μ)	3.83	3.74	
Chemical softness(S)	9.07	9.36	
Electrophilicity index (ω)	1.42	1.28	
Dipole moment	3.10	4.05	
ECT	2.56	2.57	

The Chemical softness(S) of the present molecule was appeared as 9.07 and 9.36 respectively for the both regions which was relatively high and these values ensured the reliability of the drug compound being with stable chemical reaction. Since, the Electrophilicity play important role in identifying the exchange of electronic potential whether from ligand to base or base to ligand. Here, such index was calculated to be 1.42 and 1.32 and these values are greater than unity and showed the negative potential gradient which accumulated in the molecule due to the active group of molecule. Here, the active entity part was appeared to be C=N and C–N and due to which the negative gradient was increased as such. The Dipole moment in both regions was 3.10 and 4.05 dyne respectively. It is the resultant polar activity of the molecular charges which are separated with respect to the depletion characteristics of the presence of atoms. Here, the molecule was represented by homonuclear and heteronuclear atoms and the division under the chemical reactive attractive and repulsive forces in and around the molecule declared the hetero-polar activity for the assignment of drug potency. The Electrophilicity charge transfer was measured to be 2.56 and 2.57 respectively in both regions where in which; the electronic exchange was taking place within the core rings. Since the resultant Electrophilicity charge transfer was to be -0.309, from this observation, it was confirmed that, the chemi-potential energy was definitely transferred within core rings of the present compound.

### 4.10 Polarization and hyper polarization analysis

The electronic, space charge, oriental and covalent polarization are setup in the molecule due to the molecular geometrical charge orientation in various entities of the molecule. These polarizations are induced in the compound with respect to the frequency of the absorbed radiation in which some polarization may be active and some may be nonreactive. The activeness of the polarization depends on the molecular charge depletion with respect to chemical potential generated. These polarizations are usually classified in to first order and second order polarization (hyper). As per Table 8, the first order polarization was measured in the present molecule as total and average which were found to be 219 × 10^−33^esu and 338 × 10^−33^esu respectively. These two coefficients were relatively high and the polarization was very active, which means that, the chemical reactive sites were emerged in the molecule and correspondingly persuading the drug potential. The first order hypo-active polarization is nothing but hyper active chemical potential resided over the molecule due to the cascading of action potential by the electronic accelerative energy on heteronuclear sites. The same of present case was determined to be 44.21 × 10^−33^esu and such value was moderate and this adequate to accelerate chemical potential for inducing enriched biological activity.

**Table 8:** Polarizability Δα (esu), and the first hyperpolarizability β(esu) of Beta Carboline.

Parameters	B3LYP/6-31++G(d,p)	Parameters	B3LYP/6-31++G(d,p)
α_xx_	-83.60	β_xxx_	78.2
α_xy_	2.43	β_xxy_	9.05
α_yy_	-60.29	β_xyy_	4.32
α_xz_	-0.00	β_yyy_	19.97
α_yz_	-0.00	β_xxz_	-0.0003
α_zz_	-80.83	β_xyz_	0.0001
α_tot_	219.35	β_yyz_	0.002
Δα	338.91	β_xzz_	4.95
μ_x_	-2.89	β_yzz_	2.77
μ_y_	-1.17	β_zzz_	0.0
μ_z_	0.0003	β_tot_	44.2
μ	3.12		

### 4.11 NBMO electronic-transition analysis

The configurational space boundary of electronic system of the chemical compound is usually classified in to Lewis donor and non-Lewis acceptor orbital sequence in which the entire electronic transitions of non bonding molecular orbitals are scheduled [38]. In the dynamic part of electronic energy system (non bonded orbitals), the exchange of energy was measured in terms of transitional energy among different orbitals. The chemical energy of transitions taking place between different entities of the molecule which was measured and they were tabulated in Table 9.

**Table 9:** Calculated NBMO of β-Carboline by second order Perturbation theory.

Donor (i)	Type of bond	Occupancy	Acceptor (j)	Type of bond	E2 kcal/mol	Ej – Ei au	F(I j) au
C1–C2	π	1.98	C3–C7	π*	3.02	1.16	0.053
	π		C3–C4	π*	11.37	0.32	0.056
	π		C5–N21	π*	9.91	0.28	0.048
C1–H13	σ	1.98	C2–C3	σ*	4.33	0.95	0.057
C1–N21	σ	1.98	C5	σ*	3.86	1.63	0.071
	σ		C5–H15	σ*	2.80	1.12	0.050
	σ		C11–C12	σ*	12.25	4.76	0.216
C3–C4	π		C1–C2	π*	10.09	0.31	0.051
	π		C5–N21	π*	13.81	0.29	0.057
	π		C6–C7	π*	7.45	0.36	0.047
C5–H15	σ	1.98	C1–N21	σ*	6.37	0.89	0.067
C5–N21	π	1.98	N21	π*	3.09	2.11	0.072
	π		N21	π*	4.33	2.67	0.096
	π		C1–C2	π*	11.02	0.34	0.055
	π		C3–C4	π*	7.38	0.36	0.048
C6–C7	π		C3–C4	π*	11.25	0.32	0.055
	π		C8–C10	π*	10.42	0.31	0.051
	π		C11–C12	π*	10.21	0.30	0.050
C8–C10	π		C5	π*	8.45	3.27	0.149
			C5	π*	3.87	2.54	0.089
			C7	π*	3.21	1.80	0.068
			N21	π*	4.20	2.46	0.091
			C3–C7	π*	3.36	1.09	0.054
			C8–H9	π*	12.04	1.21	0.108
			C11–C12	π*	56.97	4.74	0.465
			C6–C7	π*	6.82	0.35	0.045
C8–C10	π		C11–C12	π*	10.25	0.29	0.049
C11–C12	π		C6–C7	π*	8.77	0.35	0.051
	π		C8–C10	π*	9.71	0.30	0.049
C11–H18	σ	1.98	C6–C12	σ*	4.80	0.93	0.060
C12–H19	σ	1.98	C10–C11	σ*	4.90	0.92	0.060
C3	LP	1.99	C4	N*	2.81	11.12	0.158
C5	LP	1.99	N21	N*	7.52	11.34	0.261
	LP		N21	N*	22.24	11.91	0.459
	LP		C11–C12	π*	26.41	14.18	0.547
C7	LP	1.99	C6	N*	3.11	11.08	0.166
N20	LP	1.99	C3–C4	π*	13.41	0.39	0.066
	LP		C6–C7	π*	7.98	0.42	0.053
	LP		C11–C12	π*	5.52	4.41	0.147
N21	LP	1.99	C5	N*	4.31	1.44	0.072
	LP		C1–C2	π*	5.39	0.95	0.065
	LP		C4–C5	σ*	10.79	0.73	0.080
	LP		C5–H15	σ*	3.74	0.78	0.049
C1–C2	π	0.014	C3–C4	π*	57.37	0.02	0.066
C3–C4	π	0.016	C6–C7	π*	43.45	0.03	0.060
C5–N21	π	0.006	C1–C2	π*	49.38	0.02	0.059
	π		C3–C4	π*	38.63	0.04	0.068
C6–C7	π	0.017	C11–C12	π*	5.22	4.00	0.330
C8–C10	π	0.008	C6–C7	π*	26.66	0.05	0.066
C11–C12	π	0.008	C6–C7	π*	22.49	0.05	0.062

In the present case, the transition was observed from C1–C2 to C3–C4 with absorption of energy; 11.37 kcal/mol which was assigned to π- π* in pyridine core. In same ring, the transition was taking place from C1–N21 to C11–C12 and from C3–C4 to C1–C2 and C5–N21 by taking energy of 12.25 and 10.9 & 13.81 kcal/mol and these were assigned to be σ- σ* and π- π* interaction system. The huge energy of 56.97 kcal/mol was transferred from C8–C10 to C10–C12 in the π-π* interactive system. In the case lone pair orbitals, the transition was observed from C5 to N21 and C11–C12 by up taking energy of 22.24 and 26.41 kcal/mol in lone pair and π-π* interactive overlapping system which was moderate that showed the active presence of the drug property fabrication. In the same ring, the transitions from C1–C2 to C3–C4 was taking place by taking energy of 57.37 kcal/mol which was redirected to C6–C7 with the energy; 43.45 kcal/mol in the π- π* overlapping orbital. The important segmental energy of 49.38 kcal/mol and 38.63 kcal/mol observed from C5–N21 to C1–C2 and C3–C4 in π- π* interacting orbital system. These energies were so high and these were also the part of the process in which the considerable energy was exchanged for organizing antibiotic influence. From these transitions, it was clear that, the transitions with huge amount of energy were taking place only in the pyridine ring. From this observation, it was concluded that, the significant exchange of electronic energy was found only in pyridine ring which was gathered the energy from indole ring and organize the resultant antibiotic potential in the compound.

### 4.12 VCD examination

**Fig. 10:**
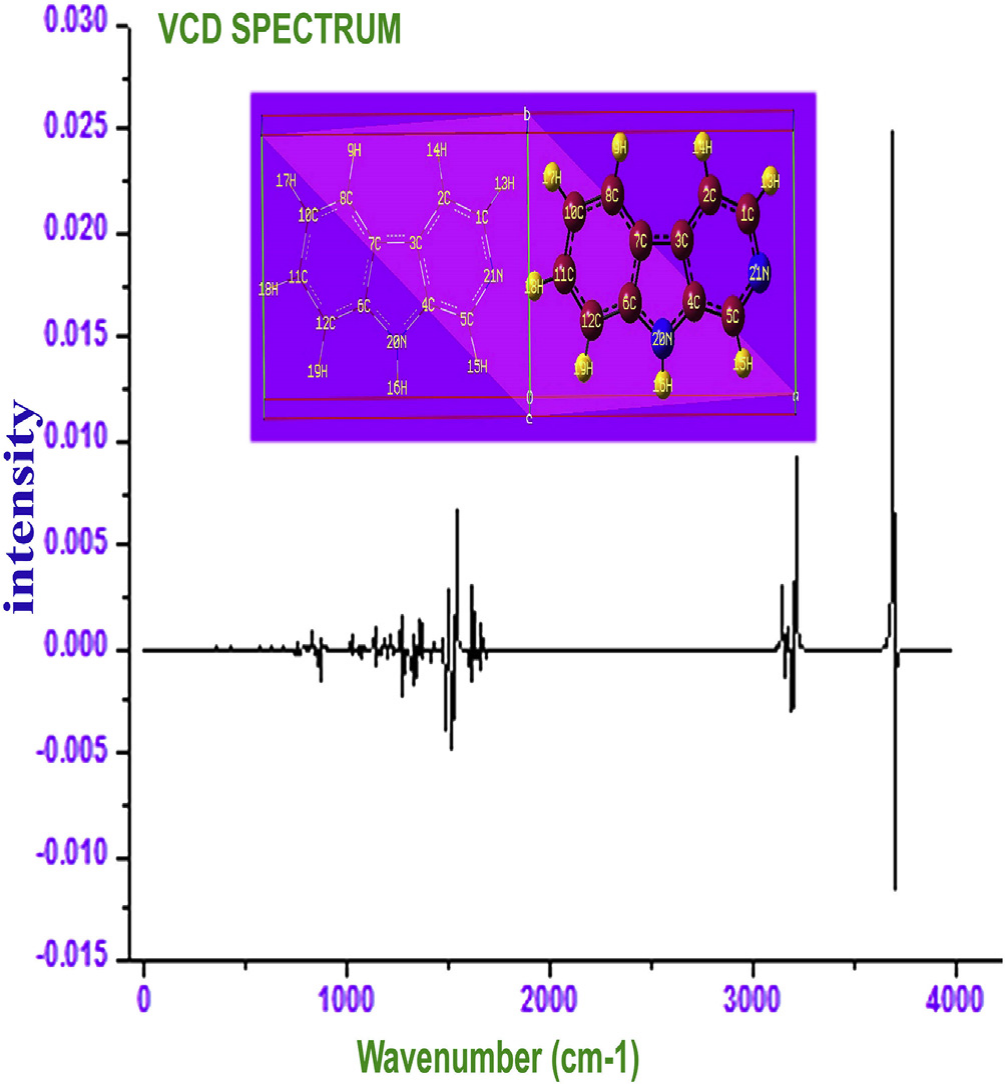
VCD profile display of Beta Carboline.

The chirality character of the chemical compound is the exposure of hindrance of toxic level and from the chiral sequence, the purity and addition of toxicity can be studied [39]. The chirality of the compound is usually studied from the vibrational circular dichroism spectrum of the chemical species. The VCD spectrum of present drug case was displayed in Fig. 10. In this case, the absorption of left and right circularly polarized lights were merged and displayed according to the spectral region of IR and Raman. Here, in the present case, the equal absorption pattern was obtained from dichroism spectra which showed no considerable noxious effect found on N–H as well as C–H allied vibrations. In mid range of IR region, fairly sequential vibrational pattern was come into sight that enabled the less toxic effect on C=N and its allied bond lengths. At far IR region, there were fluctuated absorption pattern was found which showed little bit of toxic nature in the compound. This can be removed from the molecule by optimize the structure further.

## 5 Conclusion

The antibiotic drug; β-Carboline was characterized by spectroscopically and theoretically against its biological, physico-chemical and structural properties. The structural and physico-chemical parameters proved the drug activity. The entire drug estimating factors belongs to Lipinski five rule were verified with calculated data for proving the drug quality and potential. The vibrational properties have been carried and the contribution of the compositional parts of the molecule for the drug activity. The NMR chemical shifts were observed for core and allied carbons in the compound through which the reaction path was identified and the cause of drug activity in the compound was recognized. The 6.61 eV energized orbital interaction was observed and described the drug activity consistence. The hyper active polarizability and hyperpolarizability of the compound was noted from which chemical dynamic response for obtaining antibiotic process was keenly observed. The excited electronic transitional pattern was obtained and the important transitions were evaluated and CT complex of drug activity was determined. The VCD profile was checked for finding toxicity level in the antibiotic drug and the ignorance of the contamination was evaluated. By carry out the characterization on the present drug, the quality, purity, biological affinity, structural reliability and unknown physico-chemical parameters were determined.

## Declarations

### Author contribution statement

S.Ramalingam: Conceived and designed the experiments.

R. Aarthi: Performed the experiments.

K. Hemachandran: Analyzed and interpreted the data; Wrote the paper.

P Anbusrinivasan, C.K. Nithya, Contributed reagents, materials, analysis tools or data.

### Funding statement

This research did not receive any specific grant from funding agencies in the public, commercial, or not-for-profit sectors.

### Competing interest statement

The authors declare no conflict of interest.

### Additional information

No additional information is available for this paper.
